# Supplementary figures and images for: Resequencing and characterization of the first Corynebacterium pseudotuberculosis genome isolated from camel
Source: PeerJ. 2024 Jan 30;12:e16513. doi: 10.7717/peerj.16513 (PMC10836205; doi:10.7717/peerj.16513)

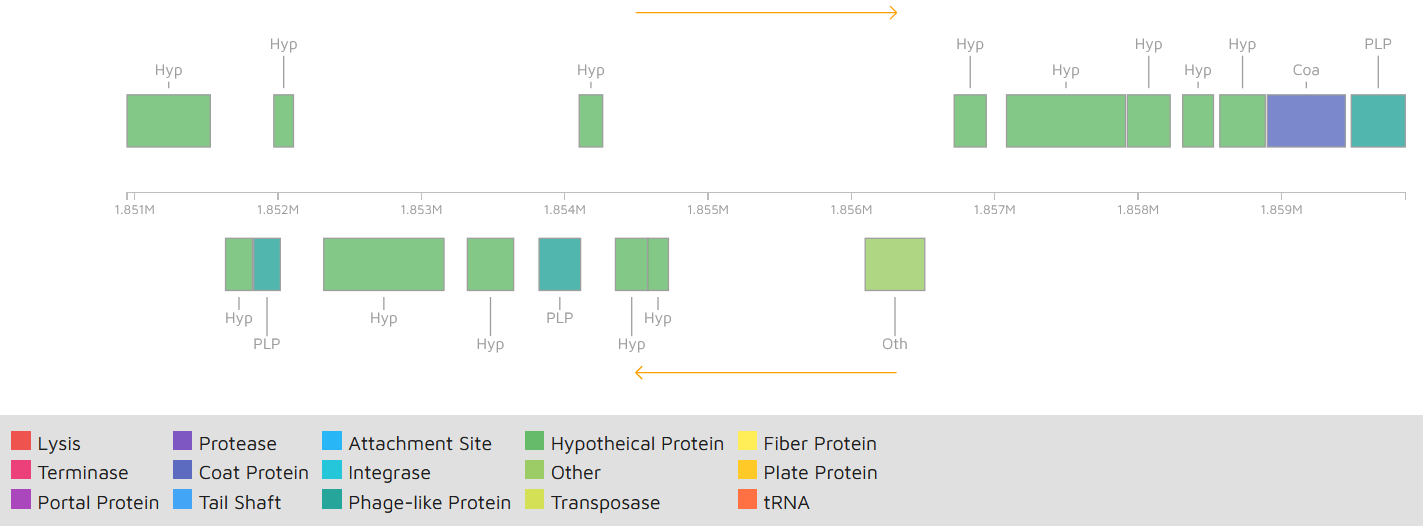

Supplement: Supplemental Information 5 [file peerj-12-16513-s005.png]
